# “Prevalence of Peri‐Implant Diseases in Patients With Type 2 Diabetes Mellitus: A Cross‐Sectional Study”

**DOI:** 10.1002/cre2.70352

**Published:** 2026-04-26

**Authors:** Luca Sbricoli, Gloria Feltracco, Francesco Cavallin, Dario D'Arienzo, Serena Bergamin, Edoardo Stellini, Eriberto Bressan

**Affiliations:** ^1^ Department of Neurosciences School of Dentistry, University of Padua Padua Italy; ^2^ Independent Statistician Solagna Italy; ^3^ Department of Odontostomatology Castelfranco Veneto Hospital, ULSS 2 Marca Trevigiana Castelfranco Veneto Italy

**Keywords:** diabetes mellitus, peri‐implant disease, peri‐implant mucositis, peri‐implantitis

## Abstract

**Objectives:**

Type 2 diabetes mellitus (T2DM) is a major systemic disease, predisposing patients to inflammatory conditions, and it is considered a grade modifier of periodontitis. However, its association with peri‐implant diseases is still under investigation. This study aimed to investigate the possible association between diabetes and peri‐implant disease.

**Material and Methods:**

Seventy patients (35 with T2DM and 35 non‐diabetic) were included, for a total of 227 dental implants in function for at least 1 year. Patient characteristics, implant features, and peri‐implant diseases (including peri‐implant mucositis and peri‐implantitis) were recorded.

**Results:**

The overall prevalence of peri‐implant diseases was not statistically different between diabetic and non‐diabetic subjects (80% vs. 77%, *p* = 0.99). When distinguishing between peri‐implant mucositis and peri‐implantitis, the prevalence of peri‐implant mucositis (51% in diabetic patients vs. 63% in non‐diabetic individuals; *p* = 0.47) and peri‐implantitis (51% in diabetic patients vs. 43% in non‐diabetic individuals; *p* = 0.63) did not differ significantly between the two groups.

**Conclusions:**

Our findings did not suggest different occurrence of peri‐implant mucositis and peri‐implantitis in diabetic and non‐diabetic subjects. Therefore, type 2 diabetes may not play a relevant role in peri‐implant diseases.

**Abbreviations**
AGE: advanced glycation end‐products
BoP: bleeding on probing
EFP: European Federation of Periodontology
IL‐1: interleukin‐1
IL‐6: interleukin‐6
IL‐8: interleukin‐8
IQR: interquartile range
PI: plaque index
PPD: pocket probing depth
RAGE: receptor for advanced glycation end‐products
T2DM: type 2 diabetes mellitus
TNF: tumor necrosis factor

## 1 Introduction

Over the past two decades, dental implants have become widely used therapeutic procedures in replacing missing teeth. Many studies with a follow‐up of at least 9 years, in fact, reported a high survival rate that contributed to recognition of dental implants as an effective rehabilitation method (Roos‐Jansåker et al. 2006a, 2006b). Moreover, the development of new surgical and prosthetic techniques considerably expanded the indication for dental implants. Although implant therapy is generally perceived as a highly satisfactory rehabilitative procedure in terms of function, aesthetics, and long‐term success (Berglundh et al. 2002), infections such as peri‐implant mucositis and peri‐implantitis are commonly observed, and their treatment will be an increasingly important part of future dental practice. Similar to periodontal diseases, peri‐implant mucositis and peri‐implantitis are considered inflammatory diseases of infectious origin that ultimately lead to the loss of peri‐implant bone support. Peri‐implant mucositis is defined as an inflammatory lesion limited to soft tissue (Heitz‐Mayfield and Salvi 2018), whereas peri‐implantitis also involves bone tissue (Berglundh et al. 2018). The management of these biological complications is essential for all clinicians who wish to achieve successful implant therapy.

Diabetes mellitus is a common chronic condition with serious health implications, and it is one of the most important and widespread chronic diseases. Epidemiologically, diabetes is a growing disease. In fact, the global prevalence of diabetes was estimated at 9.3% (463 million people) in 2019, expected to increase to 10.2% (578 million) by 2030 and to 10.9% (700 million) by 2045 (Saeedi et al. 2019). It encompasses a heterogeneous group of metabolic disorders characterized by defects in insulin production, insulin action, or both, leading to abnormal glucose metabolism, resulting in hyperglycemia. This characterizes both major types of diabetes (type 1 and type 2) (American Diabetes Association 2009). Diabetes mellitus is associated with several acute and chronic complications, and can eventually affect all organs of the body, including periodontal tissues. In fact, there is a bidirectional relationship between diabetes and periodontal disease, as diabetes is considered among the main risk factors for periodontitis, and periodontal disease is considered the sixth most frequent complication of diabetes (Löe 1993). This occurs because diabetes impairs wound healing due to an exacerbated immune response to pathogens, releasing inflammatory mediators (IL‐1, IL‐6, and IL‐8) and tumor necrosis factor (TNF‐ɑ) into oral tissues (Moura et al. 2019). Hyperglycemia leads to the accumulation of advanced glycation end products (AGEs) and it results in an increased expression and activation of their primary receptor (RAGE) (Khalid et al. 2022). AGEs may have a direct effect on the cellular phenotype via the receptor‐independent pathway, but the AGE–RAGE interaction negatively influences cellular phenotype and function (Khalid et al. 2022). This results in increased inflammation due to reduced function of neutrophil granulocytes, production of reactive oxygen species, or oxidative stress, altered collagen homeostasis associated with hyperglycemic states, and impaired tissue repair (Moura et al. 2019). Moreover, hyperglycemia directly promotes oxidative stress, and both inflammation and oxidative stress can contribute to further AGE formation (González et al. 2023). These mechanisms, combined with the effect of periodontal pathogens, perpetuate this vicious cycle of inflammatory stress and deranged repair in the diabetic periodontium. Inflammation and oxidative stress, therefore, amplify each other and can also promote changes in the subgingival biofilm. All these complex pathways result in accelerated tissue destruction in diabetic patients (González et al. 2023).

According to the proceedings of the 2017 World Workshop on the Classification of Periodontal and Peri‐implant Diseases and Conditions (Caton et al. 2018), available data on the association between peri‐implant diseases and diabetes were inconclusive and further investigations are required to address this topic (Schwarz et al. 2018).

Therefore, this study aimed to investigate the association between type 2 diabetes and peri‐implant diseases. The analogy between periodontal and peri‐implant tissues could suggest a similar dynamic to the widely demonstrated mechanism of diabetes and periodontal disease, but it may be exacerbated by the equally important differences between the two tissues that make peri‐implant sealing less effective than the periodontal one.

## 2 Methods

### 2.1 Study Design

This is a single‐center, cross‐sectional, observational study on peri‐implant diseases among diabetic and non‐diabetic patients who were treated at the Odontostomatology Department of Saint Giacomo hospital in Castelfranco Veneto (Italy) from March 15, 2024 to August 23, 2024. The study adhered to the principles of good clinical practice and was performed in accordance with the Helsinki Declaration. The study protocol was approved by the Ethics Committee of the Northern Veneto Area (CET‐ANV) (number 0005306/24), and an informed written consent was obtained from each participant.

### 2.2 Participants

Eligible subjects were patients who were at least 30 years of age, of both genders, rehabilitated with dental implants in function for at least 1 year. Patients with type 2 diabetes mellitus (T2DM) were included in the exposed group, whereas patients without diabetes were included in the unexposed group. All included patients had a pre‐existing diagnosis of diabetes, confirmed via glycated hemoglobin (HbA1c) levels retrieved from their most recent blood tests. Furthermore, all diabetic patients included in the study were on pharmacological therapy. Patients with prediabetes, metabolic syndrome or gestational diabetes mellitus, and pregnant or breastfeeding women were excluded.

**Table:** 

Parameter	Values (*n*)
Type 2 Diabetes Mellitus	35
Antidiabetic therapy:	
Diet only	2
Metformin	10
Insulin	4
Metformin + DPP‐4 inhibitor	4
Metformin + GLP‐1 receptor agonist	1
Sulfanylurea (glicazide) + GLP‐1 receptor agonist	1
Metformin + GLP‐1 receptor agonist + SGLT2 inhibitor	1
Insulin + Metformin + SGLT2 inhibitor	1
HbA1c < 7%	28
HbA1c > 7%	6
Unknown HbA1c	1

### 2.3 Procedures

Periodontal probing was performed for each patient, using a standard periodontal probe (PCP‐UNC 15, Coricama, Maniago (PN), Italy), and filling out a six‐site periodontal chart for each tooth and implant. Probing pocket depth (PPD) was measured at six sites per tooth (mesio‐buccal, mid‐buccal, disto‐buccal, mesio‐lingual, mid‐lingual, and disto‐lingual). Plaque index (PI) was recorded as the percentage of surfaces displaying plaque using the full‐mouth plaque score (FMPS). Bleeding on probing (BoP) was assessed as the percentage of sites bleeding within 15 s after probing, using the full‐mouth bleeding score (FMBS). The presence of exudation/suppuration, peri‐implant mucosal hyperplasia, and clinical mobility was also recorded. A periapical radiograph was taken for each dental implant and the marginal bone resorption was evaluated according to previous studies (Gualini et al. 2017). Briefly, radiographs were calibrated using dedicated software [ImageJ software (Wayne Rasband, US National Institute of Health, Bethesda], and the distance between the most coronal bone‐to‐implant contact and the implant shoulder was measured. As baseline radiographs were not available, marginal bone loss was calculated considering the implant shoulder as the reference point for the original bone level (*t* = 0). Any distance > 0 mm between the implant shoulder and the first bone‐to‐implant contact was recorded as percentage of bone loss both at the distal and at the mesial aspect. All clinical measurements were performed by a single experienced operator (G.F.).

### 2.4 Outcome Measures

Based on the collected data, a diagnosis of peri‐implant health, peri‐implant mucositis, or peri‐implantitis was made for each implant. The EFP S3 Clinical Practice Guidelines about prevention and treatment of peri‐implant diseases (Herrera et al. 2023) define peri‐implant health in the absence of clinical signs of inflammation and bleeding or suppuration on gentle probing, or in the presence of a single bleeding spot around the implant, in the absence of increased PPD compared to previous examinations, and in the absence of bone loss beyond crestal bone‐level changes resulting from initial bone remodeling. A diagnosis of peri‐implant mucositis is made in the presence of bleeding (more than one spot at a location around the implant or presence of a line of bleeding or profuse bleeding at any location) and/or suppuration on gentle probing, and in the absence of bone loss beyond crestal bone‐level changes resulting from initial bone remodeling, with or without increased PPD compared to previous examinations. Peri‐implantitis is diagnosed in the presence of bleeding and/or suppuration on gentle probing, increased PPD compared to previous examinations, and in the presence of bone loss beyond crestal bone‐level changes resulting from initial bone remodeling. In the present study, in the absence of previous examination data, the diagnosis of peri‐implantitis was based on the combination of the presence of bleeding and/or suppuration on gentle probing, PPDs ≥ 6 mm, and bone levels ≥ 3 mm apical of the most coronal portion of the intraosseous part of the implant.

### 2.5 Statistical Analysis

A sample size calculation was performed a priori using R 4.4 (R Foundation for Statistical Computing, Vienna, Austria) (R: The R Project for Statistical Computing n.d). Based on a previous study (Sabancı et al. 2022), 70 patients (35 diabetic and 35 non‐diabetic) were required to have an 80% chance of detecting, significant at the 5% level, a difference in the prevalence of peri‐implantitis from 8% in the unexposed group to 35% in the exposed group.

Numerical variables were summarized using median and interquartile range (IQR), whereas categorical variables were summarized using absolute and relative frequency (percentage). It is noteworthy that numerical variables were also summarized using mean and standard deviation to make future literature comparison and meta‐analysis easier (Supporting Information S1). The analysis included subject‐level variables and implant‐level variables, which were analyzed differently because the data structure included multiple implants for some subjects. Subject‐level variables were compared between two groups using the Mann–Whitney test (numerical variables) and the Chi Square test or Fisher's exact test (categorical variables). Implant‐level variables were compared between two groups using logistic regression models with cluster‐robust standard errors, as implants were nested within subjects. All tests were 2‐sided, and a *p* value of less than 0.05 was considered statistically significant. Statistical analysis was performed using R 4.4 (R Foundation for Statistical Computing, Vienna, Austria) (R: The R Project for Statistical Computing n.d).

## 3 Results

Seventy subjects (32 males and 38 females; median age 73 years, IQR 66–80) were included in the study, for a total of 235 dental implants. Eight implants were excluded from the analysis because they were not loaded (*n* = 4) or in function for less than 1 year (*n* = 4); thus, 227 dental implants were analyzed. The study sample included patients with one implant (*n* = 14), two implants (*n* = 21), three implants (*n* = 10), four implants (*n* = 10), five implants (*n* = 8), seven implants (*n* = 3), eight implants (*n* = 2), nine implants (*n* = 1), and 15 implants (*n* = 1).

Table 1 summarizes the subject characteristics and implant features in the exposed and unexposed groups. Diabetic subjects were older than non‐diabetic subjects (median 77 vs. 71 years, *p* = 0.004), whereas the other subject characteristics and implant features were not statistically different between the two groups (Table 1).

**TABLE 1:** Overview of subject characteristics and implant features in the exposed and unexposed groups.

Variables	Exposed group	Unexposed group	*p* value
	Diabetic subjects (*n* = 35)	Non‐diabetic subjects (*n* = 35)	
Sex: *n* (%)			0.09
Male	15 (43%)	23 (66%)	
Female	20 (57%)	12 (34%)	
Age: median (IQR)	77 (71–83)	71 (63–75)	0.004
Smokers: *n* (%)	1 (3%)	4 (11%)	0.36
History of periodontitis: *n* (%)	29 (83%)	33 (94%)	0.26
Periodontal diagnosis:			—
Stage 1 grade A	0/29 (0%)	1/33 (3%)	
Stage 1 grade B	0/29 (0%)	2/33 (6%)	
Stage 1 grade C	1/29 (3%)	0/33 (0%)	
Stage 2 grade A	0/29 (0%)	1/33 (3%)	
Stage 2 grade B	2/29 (7%)	6/33 (18%)	
Stage 2 grade C	3/29 (10%)	0/33 (0%)	
Stage 3 grade B	1/29 (3%)	7/33 (21%)	
Stage 3 grade C	9/29 (31%)	11/33 (33%)	
Stage 4 grade B	0/29 (0%)	2/33 (6%)	
Stage 4 grade C	13/29 (45%)	3/33 (9%)	
Supportive peri‐implant therapy	29/29 (100%)	33/33 (100%)	—
	Dental implants (*n* = 104)	Dental implants (*n* = 123**)**	
Type of prosthesis: *n* (%)			0.17
Screw‐retained	36 (35%)	25 (20%)	
Cement‐retained	68 (65%)	98 (80%)	
Loading time: median (IQR)	10 (7–17)	13 (9–18)	0.17
Loading time: *n* (%)			—
< 10 years	40 (38%)	38 (31%)	
10–20 years	52 (50%)	64 (52%)	
> 20 years	12 (12%)	21 (17%)	
Marginal bone loss, %: median (IQR)	0 (0–27)	0 (0–27)	0.40
Pocket probing depth, mm: median (IQR)	3.0 (2.2–4.0)	3.2 (2.5–4.2)	0.64
Bleeding on probing: *n* (%)	72 (69%)	78 (63%)	0.57

*Note:* Subject characteristics were compared between diabetic and non‐diabetic groups using the Mann–Whitney test (numerical variables) and the Chi Square test or Fisher's exact test (categorical variables). Implant features were compared between diabetic and non‐diabetic groups using logistic regression models with cluster‐robust standard errors, as implants were nested within subjects.

Overall, peri‐implant diseases were found in 55 subjects, including peri‐implant mucositis (*n* = 15), peri‐implantitis (*n* = 22), or both (*n* = 18). The prevalence of peri‐implant diseases was not statistically different between diabetic and non‐diabetic subjects (80% vs. 77%, *p* = 0.99; Table 2). Similarly, the prevalence of peri‐implant mucositis (51% in diabetic patients vs. 63% in non‐diabetic patients, *p* = 0.47) and peri‐implantitis (51% in diabetic patients vs. 43% in non‐diabetic patients, *p* = 0.63) was not statistically different between the two groups (Table 2). The implant‐level analysis confirmed that diabetes was not associated with peri‐implant diseases (62% in the diabetic group vs. 66% in the non‐diabetic group, *p* = 0.67), peri‐implant mucositis (28% in the diabetic group vs. 31% in the non‐diabetic group, *p* = 0.73), or peri‐implantitis (34% in the diabetic group vs. 35% in the non‐diabetic group, *p* = 0.91) (Table 2).

**TABLE 2:** Outcomes measures in the exposed and unexposed groups.

Outcome measures	Exposed group	Unexposed group	*p* value
	Diabetic subjects (*n* = 35)	Non‐diabetic subjects (*n* = 35)	
Peri‐implant disease	28 (80%)	27 (77%)	0.99
Peri‐implant mucositis	18 (51%)	22 (63%)	0.47
Peri‐implantitis	18 (51%)	15 (43%)	0.63
	Dental implants (*n* = 104)	Dental implants (*n* = 123)	
Peri‐implant disease	64 (62%)	81 (66%)	0.67
Peri‐implant mucositis	29 (28%)	38 (31%)	0.73
Peri‐implantitis	35 (34%)	43 (35%)	0.91

*Note:* Subject‐level outcome measures were compared between diabetic and non‐diabetic groups using the Mann–Whitney test (numerical variables) and the Chi Square test or Fisher's exact test (categorical variables). Implant‐level outcome measures were compared between the diabetic and non‐diabetic groups using logistic regression models with cluster‐robust standard errors, as implants were nested within subjects.

It is noteworthy that PI was higher in subjects with peri‐implant diseases (median 27%, IQR 16%–46%) compared to those without peri‐implant diseases (median 14%, IQR 8%–18%) (*p* = 0.003). It is also noteworthy that the median PI was 29% (IQR 14%–45%) in 22 subjects with only mucositis, 27% (IQR 15%–67%) in 15 subjects with only peri‐implantitis, and 24% (IQR 17%–41%) in 18 subjects with both mucositis and peri‐implantitis.

The association between peri‐implant diseases and implant features is summarized in Table 3. The occurrence of peri‐implant diseases was associated with a longer loading period of the implants (median 13 vs. 10 years, *p* = 0.02). Similarly, peri‐implant mucositis was also associated with a longer loading period of the implants (median 14 vs. 11 years, *p* = 0.04). The type of prothesis (screw‐ or cement‐retained) was not statistically associated with the occurrence of peri‐implant diseases (Table 3). A sub‐analysis of the outcome measures stratified by loading time (1–5 years, 6–10 years, and >10 years) confirmed that the occurrence of peri‐implant mucositis and peri‐implantitis was not statistically different in diabetic and non‐diabetic subjects with comparable loading time (Supporting Information S1: Table 1). A summary of numerical variables using the mean and standard deviation is also provided in Supporting Information S1.

**TABLE 3:** Outcomes measures according to implant features.

Type of prothesis, *n* (%)			
Outcome measures	Screw‐retained (*n* = 61)	Cement‐retained (*n* = 166)	*p* value
Peri‐implant disease	37 (61%)	108 (65%)	0.63
Mucositis	24 (39%)	43 (26%)	0.08
Peri‐implantitis	13 (21%)	65 (39%)	0.08
Loading time, years:			
Outcome measures	Median (IQR)		*p* value
Peri‐implant disease			0.02
No	10 (7–14)		
Yes	13 (9–19)		
Mucositis			
No	11 (7–16)		0.04
Yes	14 (10–20)		
Peri‐implantitis			0.72
No	11 (7–18)		
Yes	11 (9–18)		

*Note:* The association between implant‐level outcome measures and implant features was investigated using logistic regression models with cluster‐robust standard errors, as implants were nested within subjects.

## 4 Discussion

This cross‐sectional study aimed to investigate the association between diabetes mellitus and peri‐implant diseases. Our data did not show a different prevalence of peri‐implant disases (such as peri‐implant mucositis and peri‐implantitis) between diabetic and non‐diabetic subjects. The literature offers conflicting findings about the association between diabetes mellitus and peri‐implant diseases (Dalago et al. 2017; Wagner et al. 2022; Weinstein et al. 2020). For example, Dalago et al. investigated the risk factors for peri‐implantitis in 183 patients and 916 implants, and diabetes mellitus was not associated with increased occurrence of peri‐implantitis (Dalago et al. 2017). On the other hand, Weinstein performed a multicenter cross‐sectional study that included 248 patients and 1162 implants, and reported a significant association between diabetes and peri‐implantitis (Weinstein et al. 2020). In 2022, a systematic review on diabetes mellitus and dental implants concluded that implant‐prosthetic rehabilitation in patients with T2DM was a safe and predictable procedure (Wagner et al. 2022). In our study, no statistically significant differences were observed between diabetic and non‐diabetic subjects regarding the prevalence of peri‐implantitis (51% vs. 43%, *p* = 0.63). This finding might appear to contradict the aforementioned reports in the literature that identify T2DM as a major risk factor for peri‐implant bone loss. However, our results are consistent with more recent studies suggesting that when glycemic levels are well controlled, the biological response of peri‐implant tissues in T2DM patients is comparable to that of healthy individuals. This highlights that the quality of metabolic control, rather than the disease itself, is the key determinant for implant success. These differences may be attributed to the non‐linear and less predictable progression pattern of peri‐implant diseases compared to periodontal diseases. Additionally, there is a considerable variation in the sample sizes between the studies considered and our own, which may also contribute to the observed discrepancies.

It is noteworthy that the occurrence of peri‐implant mucositis was consistent with the available literature, but the reader may find heterogeneous reporting of peri‐implant mucositis due to the different diagnostic criteria (Atieh et al. 2013; Derks and Tomasi 2015; Lee et al. 2017). For instance, a recent systematic review reported the weighted mean prevalence of peri‐implant mucositis using the 2017 World Workshop definitions of 63%; our findings align with these ranges, considering the specific demographic characteristics of our study population (Reis et al. 2025).

In fact, Koldsland et al. highlighted how the prevalence of peri‐implantitis varied in the same subjects when changing the diagnostic criteria for peri‐implantitis (Koldsland et al. 2010). In our study, the peri‐implant classification (healthy, peri‐implant mucositis, and peri‐implantitis) was based on the definitions of the EFP S3 Clinical Practice Guideline about prevention and treatment of peri‐implant diseases (Herrera et al. 2023).

Inadequate plaque control may contribute to the development of peri‐implant diseases (Rokaya et al. 2020). In our study, subjects with peri‐implant diseases showed higher plaque index compared to healthy subjects. These findings were in agreement with a previous study reporting an association between poor plaque control and peri‐implant diseases (Ferreira et al. 2006; Konstantinidis et al. 2015).

However, contrary to Lagunov's assertion, this study did not show a statistically significant difference between the two groups in the mean peri‐implant BoP and the mean peri‐implant PPD, which are considered additional key elements for the diagnosis of an inflammatory state of the peri‐implant mucosa. With regard to the parameters mentioned above, Lagunov (Lagunov et al. 2019) reported a significant increase in BoP in the T2DM group compared to the healthy group (56% vs. 44.5% respectively), as well as a significantly higher PPD in the T2DM group. On the other hand, the prospective study by Tawil et al. (2008) (Tawil et al. 2008), similar to the present study, showed no noteworthy difference in peri‐implant PPD between patients with controlled diabetes and non‐diabetic patients, although it showed a statistically significant difference in the mean values of peri‐implant BoP between patients with decompensated diabetes and systemically healthy patients. Therefore, this suggests that in patients with diabetes, the progression of peri‐implant disease is greater than that in non‐diabetic patients, despite the development frequency of peri‐implant disease being superimposable in the two groups.

Several crucial factors may contribute to the success of implant therapy, including the choice between screw‐ and cement‐retained implant rehabilitation (Fiorillo et al. 2024). Specifically, the cemented prosthesis may be prone to the development of peri‐implantitis due to cement remnants (Wilson 2009), and previous studies showed higher occurrence of peri‐implantitis in cement‐retained implants (Dalago et al. 2017; Weinstein et al. 2020; Marrone et al. 2013). Our data indicated a higher occurrence of peri‐implantitis in cement‐retained implants (39%) compared to screw‐retained ones (21%). This finding finds biological plausibility in the literature: excess cement remnants can act as a rough substrate that facilitates bacterial biofilm accumulation and is often difficult to remove, triggering a persistent inflammatory response in deeper peri‐implant tissues (Staubli et al. 2017). Conversely, the slight trend of screw‐retained implants toward mucositis (39% vs. 26%) might be related to the micro‐gap at the implant–abutment interface, which may induce more superficial soft tissue inflammation (Reis et al. 2025).

Another contributing factor for implant success is the period of functional loading (Rokaya et al. 2020; Pimentel et al. 2018). In our study, peri‐implant disease—specifically peri‐implant mucositis—was associated with a longer period of functional loading. Similarly, Marrone et al. reported higher occurrence of peri‐implant disease—specifically peri‐implantitis—in implants in function for over 10 years (Wilson 2009).

A relevant finding of our study is the high prevalence of patients with a history of periodontitis in both the diabetic (83%) and non‐diabetic (94%) groups. It is well established in the literature that a history of periodontitis represents a significant risk indicator for the development of both peri‐implant mucositis and peri‐implantitis (Ferreira et al. 2018; Serroni et al. 2024). In our cohort, this high baseline prevalence of periodontal disease may have acted as a confounding factor, potentially masking the specific impact of type 2 diabetes on peri‐implant tissues. As most subjects in both groups were already “at risk” due to their periodontal history, the additional inflammatory burden of diabetes might not have resulted in a statistically significant difference in peri‐implant disease prevalence. This underscores the importance of strict supportive periodontal therapy in these patients, which was indeed performed for all subjects with a history of periodontitis in our study.

This study has some limitations that should be considered. First, the cross‐sectional design precludes any considerations about the causal relationship between diabetes and peri‐implant diseases. Second, the small sample size warrants caution in the interpretation of the findings. The sample size of the study was estimated a priori according to information from a previous study. Surprisingly, the data showed a prevalence of peri‐implantitis that was higher than expected and very close between diabetic and non‐diabetic subjects; hence, the study was underpowered to detect the actual difference between these groups. Furthermore, no calibration of the examiner nor use of controlled probing force was adopted for this study. Despite these limitations, we believe that our findings contribute toward addressing the gap in knowledge on the topic, as previously mentioned (Caton et al. 2018; Schwarz et al. 2018).

## 5 Conclusions

In conclusion, our findings suggest that implant rehabilitation is a safe and predictable procedure in patients with type 2 diabetes, as they did not show a significantly higher prevalence of peri‐implant diseases compared to non‐diabetic subjects. However, given the limitations of the present study, further prospective research with larger cohorts and standardized clinical protocols is warranted to confirm these observations and to better define long‐term maintenance strategies for this patient population.

## Author Contributions

Conceptualization: Luca Sbricoli and Gloria Feltracco. Methodology: Dario D'Arienzo. Formal analysis: Francesco Cavallin. Investigation: Gloria Feltracco and Serena Bergamin. Data curation: Francesco Cavallin. Writing – original draft preparation: Eriberto Bressan, Gloria Feltracco and Luca Sbricoli. Writing – review and editing: Francesco Cavallin and Eriberto Bressan. Visualization: Dario D'Arienzo. Supervision: Edoardo Stellini. Project administration: Edoardo Stellini. All authors have read and agreed to the published version of the manuscript.

## Funding

The authors have nothing to report.

## Ethics Statement

The study adhered to the principles of good clinical practice and was performed in accordance with the Helsinki Declaration. The study protocol was approved by the Ethics Committee of the Northern Veneto Area (CET‐ANV) (number 0005306/24), and an informed written consent was obtained from each participant.

## Consent

All participants provided written informed consent, which included permission for the anonymized use of their clinical data for publication. No identifiable personal information is included in this manuscript.

## Conflicts of Interest

The authors declare no conflicts of interest.

## Supporting information


Supporting File

## Data Availability

The data sets used and/or analyzed during the current study are available from the corresponding author on reasonable request.
