# Supplementary material for: “Prevalence of Peri‐Implant Diseases in Patients With Type 2 Diabetes Mellitus: A Cross‐Sectional Study”
Source: Clin Exp Dent Res. 2026 Apr 26;12(2):e70352. doi: 10.1002/cre2.70352 (PMC13110833; doi:10.1002/cre2.70352)
Supplement: Supplementary file 1 — Supporting File [file CRE2-12-e70352-s001.docx]

**SUPPLEMENTARY MATERIAL**

Supplementary Table 1: Outcomes measures in exposed and unexposed groups stratified by loading time

| Outcome measures | Exposed group (diabetic subjects) | Unexposed group (non-diabetic subjects) | p-value |
| --- | --- | --- | --- |
|  | **Dental implants with loading time 1-5 years (n=15)** | **Dental implants with loading time 1-5 years (n=7)** |  |
| Peri-implant disease | 7 (47%) | 1 (14%) | 0.26 |
| Peri-implant mucositis | 7 (47%) | 1 (14%) | 0.26 |
| Peri-implantitis | None | None | - |
|  | **Dental implants with loading time 6-10 years (n=40)** | **Dental implants with loading time 6-10 years (n=34)** |  |
| Peri-implant disease | 22 (55%) | 22 (65%) | 0.60 |
| Peri-implant mucositis | 4 (10%9 | 7 (21%) | 0.16 |
| Peri-implantitis | 18 (45%) | 15 (44%) | 0.96 |
|  | **Dental implants with loading time >10 years (n=49)** | **Dental implants with loading time >10 years (n=82)** |  |
| Peri-implant disease | 35 (71%) | 58 (71%) | 0.95 |
| Peri-implant mucositis | 18 (37%) | 30 (37%) | 0.99 |
| Peri-implantitis | 17 (35%) | 28 (34%) | 0.97 |

Supplementary Table 2: Overview of subject characteristics and implant features: summary of numerical variables as mean and standard deviation (SD)

| Variables | Exposed group | Unexposed group |
| --- | --- | --- |
|  | **Diabetic subjects (n=35)** | **Non-diabetic subjects (n=35)** |
| Age, years: mean (SD) | 75 (9) | 69 (10) |
|  | **Dental implants (n=104)** | **Dental implants (n=123)** |
| Loading time: mean (SD) | 12 (6) | 14 (7) |
| Marginal bone loss, %: mean (SD) | 17 (27) | 12 (18) |
| Pocket probing depth, mm: mean (SD) | 3.6 (1.8) | 3.4 (1.2) |

Supplementary Table 3: Summary of loading time as mean and standard deviation (SD)

| Outcome measures | Mean (SD) |
| --- | --- |
| Peri-implant disease:  No  Yes | 10 (6)  14 (7) |
| Mucositis:  No  Yes | 12 (6)  15 (8) |
| Peri-implantitis:  No  Yes | 12 (7)  13 (6) |
